# Parent-identified opportunities for improving asthma care for children insured by Medicaid following implementation of statewide Medicaid Accountable Care Organizations in Massachusetts

**DOI:** 10.3389/falgy.2025.1695447

**Published:** 2025-11-24

**Authors:** Sarah L. Goff, Charlotte F. Gilson, Sai S. Chilakapati, Joyce Mogaka, Berry L. Williams, Erin DeCou, Kimberley H. Geissler

**Affiliations:** 1 Department of Health Promotion and Policy, School of Public Health & Health Sciences, University of Massachusetts Amherst, Amherst MA, United States; 2 Department of Healthcare Delivery and Population Sciences, University of Massachusetts-Chan Medical School—Baystate, Springfield, MA, United States

**Keywords:** pediatric, primary care, asthma, qualitative, medicaid, ACO, health care reform

## Abstract

**Background:**

Childhood asthma is common and associated with extensive racial, ethnic and socioeconomic healthcare inequities and health disparities. Approximately 50% of children with asthma are insured by Medicaid in the U.S. and states have increasingly implemented Accountable Care Organization (ACO) models in their Medicaid programs, but little is known about the effects of ACOs on pediatric asthma quality of care, utilization, and disparities. Seventeen new ACOs were implemented in Massachusetts in 2018. Delivery System Reform Incentive Payments were provided to ACOs that could be used to improve outcomes for chronic diseases, such as asthma, through quality measures, enhanced care coordination, and community health worker staffing. This qualitative study explored caregiver experiences with pediatric asthma care for their Medicaid-insured child following ACO implementation in Massachusetts.

**Methods:**

Semi-structured virtual interviews were conducted with caregivers of Medicaid-insured children with asthma in Massachusetts between July 1-December 31, 2023. Purposive sampling aimed to include a range of participant and practice characteristics. The overarching theoretical framework was an adaptation of the Framework of Asthma Disparities, and data were analyzed using rapid qualitative analytic methods.

**Results:**

Of the 26 participants, 96% were female; 23% identified as Black and 39% as Hispanic. Key themes included: (1) Perceived lack of changes in asthma care related to Medicaid ACO implementation; (2) Insurance coverage influences on asthma care; (3) Perceptions of asthma management in primary care; (4) Perceptions of asthma specialist care; (5) Influence of health related social needs on pediatric asthma care and outcomes; and (6) Suggestions for improving pediatric asthma care in Medicaid ACOs. Continuity of care, communication, and asthma education were prominent subthemes.

**Conclusions:**

Medicaid ACOs efforts to transform care delivery through increased resources and improved infrastructure for care coordination and other aspects of care may not have had a substantial influence on asthma care for children in early years of implementation, addressing a gap in knowledge about mixed-age ACOs' effects on pediatric populations. Participants' perceptions of the importance of care continuity, specialty access, and education may warrant further exploration in general and in the context of Medicaid ACO effects on asthma care for children at high risk for asthma disparities.

## 1 Introduction

Asthma is one of the most common chronic diseases among children (1) and a leading cause of preventable emergency department (ED) visits, hospitalizations, school absences, and lost caregiver workdays in the U.S. (2). Socioeconomic and racial/ethnic disparities in asthma prevalence, care quality, and outcomes have persisted for decades despite efforts to address these issues (3–6). Although multi-level (e.g., health care, school, home) interventions that take into account the socially determined inequities that drive disparities (e.g., home environment, poor quality health care) have been efficacious in experimental settings (7, 8), asthma remains uncontrolled in nearly 50% of children in the U.S. (9).

Accountable care organizations (ACOs) aim to improve healthcare value through improved quality of care, decreased costs, and improved patient experience. ACOs incentivize care integration and population health through shared risk contracts and payment models designed to promote team-based care management and coordination. Shared risk contracts with upside and downside risk mean that all entities participating in an ACO contract are eligible to receive a financial benefit for successfully increasing quality of care and/or lowering costs (upside risk) and share in financial losses if quality of care is not adequate and/or costs are not contained (downside risk) (10). This value-based care model is intended to offer advantages over traditional fee-for service models for addressing complex inequities in healthcare and disparities in health, such as through systematic assessment of and response to health-related social needs (HRSN) (11). Medicaid insures more than half of children with asthma in the U.S. (12), most of whom are at higher risk for poor and disparate asthma outcomes. Medicaid programs in 14 states have implemented ACOs and the Centers for Medicare and Medicaid Services (CMS) aims to have all Medicaid programs include ACOs (13). The ACO model has shown promise for improving some chronic disease outcomes for adults (14) and in some pediatric-only ACOs (15). However, incentives for pediatric care in earlier ACO-like health care reform in Massachusetts did not appear to have an impact on pediatric quality of care (16) and data on the effects of Medicaid ACOs on pediatric care are limited (17).

Massachusetts, a state with high pediatric asthma rates and racial, ethnic and socioeconomic disparities (18), launched 17 new Medicaid ACOs in 2018 under a CMS Section 1,115 Waiver, most with shared risk contracts. The ACOs received Delivery Service Reform Incentive Program (DSRIP) funds to invest in infrastructure to transform healthcare delivery though initiatives such as technology improvements, expansion of team-based care coordination and management, and systematically assessing and addressing HRSN (19). The ACOs had a mix of shared risk profiles, but all ACO practices were held accountable for a core set of ACO quality measures, including the Asthma Medication Ratio. Most practices would also have been responsible for other asthma quality measures commonly assessed by other insurers, such as hospitalization and ED visit rates, ED follow-up, and routine asthma visits. All ACOs also had access to Flexible Spending Plan funds that could be used to improve housing, which, in turn, can decrease risk for environmental asthma triggers. By 2019, more than 90% of eligible children received care in a Medicaid ACO in Massachusetts (20). Several quantitative studies have examined the impacts of Massachusetts Medicaid ACOs overall and on children with asthma. Sabatino and colleagues found a decline in rates of pediatric asthma admissions (20), while a quantitative study conducted by our team found persistent insurance-based disparities in routine asthma visit rates and increases in ED and hospitalization rates for Medicaid-insured children with asthma following Medicaid ACO implementation (21). Understanding caregivers' experiences with asthma care following Medicaid ACO implementation, particularly in aspects of care prioritized in the Massachusetts Medicaid ACOs provides a more complete understanding of real-world experiences with pediatric asthma care in the years following ACO implementation.

The current study aimed to qualitatively characterize caregivers' (parents and guardians) overall experiences with asthma care for their Medicaid-insured children in Massachusetts, to understand their perceptions of changes in asthma care since Medicaid ACO implementation, and to elicit recommendations for improving asthma care in these ACOs.

## 2 Methods

**Figure 1:**
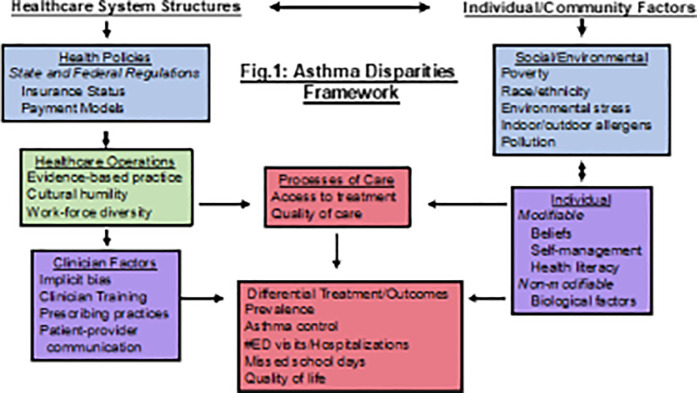
Framework of asthma disparities. Adapted from Canino et al. (22)

The conceptual framework for the overall study (quantitative and qualitative elements) was adapted from Canino and colleagues' Framework of Asthma Disparities (Figure 1) (22). Data collection focused on understanding the potential effects of the newly implemented Medicaid ACOs on pediatric asthma quality of care, utilization, and disparities at multiple organizational levels. The Consolidated Framework for Implementation Research (CFIR) (23) was used to develop the interview guide, as CFIR's multi-level domain structure (i.e., Intervention Characteristics, Outer Setting, Inner Setting, Characteristics of Individuals, and Process) mirrors and complements the multi-level organization of the Framework of Asthma Disparities Furthermore, the CFIR framework allows for identifying factors at multiple levels that may inhibit or promote successful implementation and outcomes of broad policy changes, such as the changes in health care finance and delivery associated with the ACOs.

Semi-structured virtual interviews were conducted between July and December 2023 with a purposive sample of caregivers of children with asthma who were insured by Medicaid and received primary care in a Massachusetts Medicaid ACO practice. Overall sampling aimed to include: (1) participants with a range of demographic characteristics (e.g., race/ethnicity, primary language) and child asthma severity and (2) Medicaid ACO practices with a range of organizational characteristics (e.g., geographic location, practice size, which ACO the practice belonged to). Participants were recruited from a subset of the 17 ACOs, selected to achieve sampling goals. Recruitment strategies included posting flyers at Medicaid ACO primary care practices and community sites, such as Boys' and Girls' Clubs, and tabling at community events and primary care practices.

The interview guide was pre-tested with the first three study participants with an *a priori* plan to include the data from these interviews in the analysis. No substantial changes were made to the interview guide because of pre-testing. A research team member with expertise in qualitative methods led each interview while a second team member took field notes. Interviews lasted approximately 30–60 min, were audio-recorded with the participant's permission, and professionally transcribed verbatim. Interviews were conducted until data saturation was achieved (24). Participants were given a $50 e-gift certificate in appreciation of their time. The manuscript adheres to the Consolidated Criteria for Reporting Qualitative Research (25) and the study was approved by the University of Massachusetts-Amherst Institutional Review Board.

### 2.1 Analysis

Rapid qualitative analytic methods were used; this method has been validated through comparison with deductive in-depth qualitative analysis and applicable when there is a focused area of inquiry and a targeted use of the data (26). In Phase 1, a matrix for summarizing interview data interview grounded in CFIR domains was developed. Trained pairs of analysts summarized each interview using the matrix. Summaries included key concepts and supportive quotes for each domain. Memos were used to identify emerging themes, and the analytic approach allowed for addition of domains to the matrix that may have fallen outside of the CFIR domains. In Phase II, the analytic team consolidated interview summaries to generate pertinent broad themes and sub-themes, continuing to use memos to identify potential emerging themes. Finally, team members met to discuss the themes that had emerged during Phase II analysis and organize them into major themes and subthemes. During this phase, the relationship of the themes to the Framework of Asthma Disparities was explored.

Reflexive considerations included the following: SG is a primary care pediatrician, which may have informed her interpretation of the data; SC, CG, and JM were student research assistants in SG's research lab, potentially influencing analytic discussions in relation to power differentials despite efforts to minimize these differentials.

## 3 Results

Interviews were conducted with 26 participants from 15 practices in three ACOs. Of these participants, the majority (*n* = 14; 53.8%) were between 30 and 39 years old; 25 (96.2%) identified as female; most children were between the ages of 5–9 years old (*n* = 12; 46.2%) nine identified as White (34.6%), six as Black (23.1%) and 10 as Latino/Hispanic (38.5%) (participants could select more than one response). Four interviews were conducted in Spanish. Additional participant demographics and practice characteristics are in Supplementary Table S1.

Major themes included: (1) Perceived lack of changes in asthma care related to ACO implementation; (2) Insurance coverage influences on asthma care; (3) Perceptions of asthma management in primary care; (4) Perceptions of asthma specialist care; (5) Influence of HRSN on pediatric asthma care and outcomes; and (6) Suggestions for improving pediatric asthma care in Medicaid ACOs. Themes are described in detail below and illustrative quotes corresponding to each theme are provided in Supplementary Table S2. The adapted Framework of Asthma Disparities elements associated with each theme are shown in parentheses.

### 3.1 Perceived lack of changes in asthma care related to ACO implementation (health policies/payment models)

Participants were either not aware that their child's primary care practice was part of a Medicaid ACO, or they knew the practice was part of an ACO but did not know what that meant functionally for potential changes in care delivery. Some participants noted improvement in communication with their child's PCP or the office staff through a patient portal or by phone, and some felt communication between their child's PCP and asthma specialist had improved. Some participants also discussed being newly referred to a home-visit program focused on mitigating environmental asthma triggers. Although these changes could have been related to ACO initiatives, they also could have been related to other initiatives, such a home visiting program that had been initiated by the state department of public health.

### 3.2 Insurance coverage influences on asthma care (health policies/insurance status)

Participants generally reported satisfaction with the Massachusetts Medicaid program's coverage for their child's asthma medications, office visits with their PCP and, if relevant, an asthma specialist. However, some participants also expressed dissatisfaction with coverage for asthma-related needs, such as nebulizers, hospitalizations, and medication coverage for their child once they turned 18 years of age.

“The doctor's office was very helpful in making sure that she got the meds that she needed … But then, when it came to the MassHealth side … they kept telling me that I had to pay out of pocket for it … I don't have the money to pay out of pocket for this … I must have called them at least six times to try to get this figured out … It ended up taking a week or so for me to be able to get everything straightened out.” (03-03; Private Practice; Northeast; White; not Hispanic or Latino)

“And if I didn't have MassHealth at all, I really don't know how I would be able to afford my son's like $300 a month medication that he literally needs that he takes every single day. And so, if you're a person who is dealing with asthma and you don't even have the medication that you need because the finances and everything is expensive these days, cost of living is so expensive, it's a lot. It is a lot.” (03–05; Private Practice; Southeast; Black; not Hispanic or Latino) 

### 3.3 Perceptions of asthma management in primary care (multiple levels of the framework of asthma disparities)

Participants described factors that they felt positively and negatively influenced asthma care for their child in their PCP's practice. These factors, described in detail below, represented several elements of the Framework of Asthma Disparities.

#### 3.3.1 Patient-provider communication (clinician factors)

Some participants felt that their child's PCP communicated well about their child's asthma and fully addressed their questions, while others felt they did not receive adequate asthma education from their child's PCP or members of the primary care team. Some felt that there was not enough time in their child's appointments to ask questions. Some participants felt that their child's PCP used too many medical terms that they did not understand. For participants with limited English proficiency, some felt that inadequate translation services made caring for their child's asthma more challenging.

“[When child was first diagnosed] it was really hard…the way a doctor speaks sometimes is hard for somebody that doesn't know lingo to understand. They're not explaining it as much, they're just like, ‘okay here, give them the medicine, the nebulizer' or there's just no backup support. It's like here you have everything you need but now you have to figure out what to do with it.” (03–06; Private Practice; Western; White; not Hispanic or Latino)

#### 3.3.2 Workforce diversity (health care operations)

One participant, while discussing the ways implicit and explicit bias affected their life, shared that they felt racial discordance with their child's providers made it challenging for the provider to fully understand the challenges they face every day. They felt that although the PCP meant well, they could not fully understand the experience of someone in a minority racial group and therefore limited in their ability to address the totality of their needs.

#### 3.3.3 Continuity of care (processes of care)

Some participants expressed frustration with a lack of continuity of care with their PCP (e.g., a different provider for each visit), feeling that care was suboptimal when continuity was poor. Other participants felt that they had good continuity and that it was important for their child to see someone who knew them, their asthma history, and their current asthma care plan well, particularly when they needed an urgent visit for worsening asthma symptoms. Some participants commented on long wait times for non-urgent appointments, which diminished opportunity for non-urgent visits focused on addressing questions about asthma management and prevention of asthma exacerbations. Some caregivers expressed a desire for one point of contact in their child's primary care office for all asthma management.

“At [PCP name], they kind of give you whatever doctor is available. You don't see a consistent pediatrician … he had the same pediatrician [at previous practice]. It's not the same experience and when you have a doctor that you're consistent with, they understand that and they also understand everything else that's tied into it, like their overall health.” (03–06; Private Practice; Western; White; not Hispanic or Latino)

#### 3.3.4 Perceptions of PCP support in treating asthma (clinician factors/patient-provider communication)

Some participants felt that they had to educate themselves about asthma, including being responsible for managing their child's asthma largely on their own. Some participants expressed concern that if they did not take charge of their child's asthma management, they feared that their child would have a severe asthma exacerbation. Participants who felt this way generally wished that they could have more help from their child's doctors and did not have to feel that the responsibility for managing their child's asthma was theirs alone. Some caregivers expressed that they considered acute care facilities (e.g., emergency department) their only option when the PCP could not adequately treat their child's asthma.

“I've had to advocate so hard for my children and making sure that they receive the care that they need … If I didn't have, you know, that advocacy skill set, and I wasn't able to communicate on my own or feel confident enough to do it, I don't think that my children would receive the same amount of care … ” (03–14; Private Practice; Southeast; Black; not Hispanic or Latino) 

### 3.4 Perceptions of asthma specialist care

Participants were asked if their child currently saw or had ever seen an asthma specialist. If their child had seen a specialist, they were asked about the referral process and experience with specialist care.

#### 3.4.1 Satisfaction with care and care coordination (processes of care/ quality of care)

Participants whose child saw an asthma specialist (e.g., allergist, pulmonologist) at least once generally preferred asthma care delivered by the specialist compared to the PCP. Specific ways in which specialist care was felt to be preferable included having more time with the specialist, which allowed for development of an asthma action plan, more detailed asthma education, and better communication outside of office visits. Participants' perspectives on asthma care coordination varied. Some participants felt coordination between their child's asthma specialist's practice and PCP was poor, requiring extra communication and work by the caregiver, while others felt the communication between specialist and PCP was good or had improved in recent years.

“The first thing that [the specialist] told me was like, ‘You're not going to leave here without a plan.’ … I was so relieved because we had gone through so many sleepless nights, so many days of having to take off of work because my son's not feeling well.” (03–05; Private Practice; Southeast; Black; not Hispanic or Latino) 

“I think that [the PCP and specialist] were not working together. I think when I go to this doctor, I have to tell him … everything all over again. And then when I go to see the other one, I have to say all over everything from the beginning again … I think that's why I stopped seeing the allergy doctor. I was like, I'm not gonna see her no more.” (01–07; CHC owned by a hospital; Western; race not reported; Hispanic or Latino) 

#### 3.4.2 Access to treatment (processes of care)

Some participants were not aware that asthma specialists exist and reported that they wished that their PCPs had let them know about this resource. Some participants who sought consultation with an asthma specialist commented on struggling to obtain a referral and once referred, experiencing long wait times for the first appointment to see the specialist. Some reported that it was then not difficult to get an appointment after establishing care with the specialist.

### 3.5 Influence of HRSN on pediatric asthma care and outcomes (social/environmental)

The new Medicaid ACOs in Massachusetts were required to implement universal annual HRSN screening of all members. ACOs were given latitude to decide what screening instrument to use and how to implement the screening. ACOs received very limited new resources through the 1,115 Waiver to respond to social needs identified through screening.

#### 3.5.1 Screening

In response to questions about health-related social needs screening, some participants reported that their PCP had not asked them about health-related social needs but also did not expect this to be the PCP's responsibility. Of those who experienced health-related social needs screening, some reported receiving assistance in response to a need while others described feeling upset that there was no follow-up after divulging a need on a screening form.

#### 3.5.2 Health-related social needs effects on asthma care and outcomes

Participants shared several ways that they felt HRSN affected their child's asthma and their life. Most families qualify for Medicaid insurance because they have lower household incomes and HRSNs described by participants were generally related to limited financial and other resources. For example, poor quality rental housing with indoor environmental triggers generated stress due to an inability to move because of household income. Some participants reported having limited social support, although some said they learned about asthma management from other caregivers whose children had asthma. Transportation to doctors' visits and missed days of work due to their children's asthma contributed to caregiver stress. One participant described experiencing “environmental racism” and how they felt it negatively affected them and their child's asthma care. Although the participant did not describe what they meant by the term, environmental racism has been defined as the ways in which government policies and regulations cause a disproportionate impact of environmental hazards on Black/African American, Latin, Indigenous, Asian American and Pacific Islander, migrant farmworkers and other workers with lower incomes (27).

“There was a time when … the heaters weren't working, and that lasted for a week, and it affected my son a lot … I told [child's PCP] that, because I couldn't pay the electricity bill, and they interrupted our energy services like three times, but the last time they were going to suspend it, the doctor intervened, his pediatrician called them and explained the situation, that my child needed the electricity for his treatment … I think [child's PCP] did what he had to do, because he sent a letter to the electricity company, and he also gave my son a treatment, and told me not to worry, that he needed that treatment and I wasn't going to have the electricity cut off. And it did go that way, because they didn't cut it off when they said they would. So, he did help me.” (04-02; CHC; Western; race not reported; Hispanic or Latino)

### 3.6 Suggestions for improving pediatric asthma care

Many of the suggestions participants made about areas ACOs could focus on in the future to improve asthma care for children were related to elements of asthma care participants offered critiques of during discussion. Suggestions included improved asthma education by the PCP, particularly at the time of diagnosis, increased time during visits with PCP, transparency about existence of specialist care for asthma and indications for referral, and improved care coordination, including having one point of contact for asthma communication.

“When I first started with the asthma journey, I think it would have been helpful to have one person explain everything, instead of going from doctor to doctor. There was a lot of stuff I didn't know … I don't think people take asthma as seriously as they should … There's language barriers. Some parents just don't understand it. So maybe a more centralized, one person, one place, all the resources. I think that would be helpful.” (03-01; Private Practice; Northeast; White)

Educating parents early about what asthma looks like, what allergies look like, how they're connected [would be helpful … I would say that's the biggest [change to improve healthcare system is] educating parents early. Letting us know because asthma and allergies, they're on the rise … Tell us early so that we know what to look for and we can take action earlier on. (03–05; Private Practice; Southeast; Black; not Hispanic or Latino)

## 4 Discussion

This study explored caregivers' perceptions of changes in asthma care for their child following implementation of Medicaid ACOs in Massachusetts as well as their general experiences with their child's asthma care, aiming to identify opportunities ACOs might consider when seeking to optimize care delivery. Some caregivers noted minor changes in their child's asthma care that may or may not have been directly related to ACO investments in transforming care deliver but they identified several opportunities for improving pediatric asthma care in ACO practices. Themes identified linked to each element of the adapted Framework of Asthma Disparities and CFIR domains. Some of these opportunities for care optimization were consistent with previously identified needs, while others were more related to the context of Massachusetts' Medicaid ACOs. For example, participants desired asthma education that is understandable to a caregiver and child, that is delivered in a family's primary language, and is iterative. The Expert Panel Report 3 (EPR-3): Guidelines for the Diagnosis and Management of Asthma include asthma education as one of the four key components of care but offer little guidance on how to tailor asthma education to ensure understanding of disease processes and the rationale for management recommendations (28). Deconstructing the barriers and facilitators to adequate asthma education and testing implementation of best practices for education may be warranted. The confusion caused by frequent changes in asthma medication coverage for Medicaid patients was an example of a health policy consequence that may or may not have been experienced by caregivers in other states. Further study of the effects of formulary changes on adherence to asthma medication regimens may be warranted.

The requirement that the Massachusetts Medicaid ACOs screen members annually for HRSN could improve asthma care and outcomes (4). Although the universal screening requirement provided an important incentive to ensure screening takes place, the resources provided through the Flexible Spending Plan implemented by the Massachusetts Medicaid program to address social needs identified by screening were generally limited to addressing housing issues and food insecurity (29). Given the stigma associated with poverty in the U.S. and potential parental concerns about confidentiality of screening (30), it may be difficult for some families to disclose health-related social needs. When needs are disclosed and not recognized or are recognized but can't be addressed, screening can have unintended negative consequences (31). More research is needed to determine the most effective approaches to HRSN screening, the effects of screening in the absence of adequate resources to address identified needs, and the degree to which screening and referral can influence health disparities for conditions such as asthma in the absence of upstream policy changes that address the root causes of inequities in health care and health disparities are needed.

Several of the themes identified in the current study relate to previously described issues with care fragmentation in the U.S. health care system (32). Although the potential consequences of care fragmentation for adult populations have been described (33, 34), fewer studies have examined continuity of care in pediatric populations (35, 36), particularly in regard to fragmentation within a primary care practice. While some participants in the current study were satisfied with their child's asthma care, others felt that better continuity with the child's PCP was needed to provide optimal care. Some also desired a single point of contact in the PCP's office for asthma management. PCP-level continuity has been associated with better asthma outcomes, such as reduction in emergency department visits, and children in lower income and racial minority groups may benefit the most from PCP continuity (37). The streamlined communication and PCP continuity desired by some of the current study's participants could potentially address some of the recommendations participants made, such as delivering more effective asthma education and helping families that need more support feel like they are not fully responsible for managing their child's asthma. In addition to improved communication from PCP's, some participants felt that inclusion of an asthma specialist to their child's team was valuable for understanding and managing asthma. Prior work has shown that children with Medicaid are less likely to receive specialist care than privately insured children (38). This means that ensuring that Medicaid-insured children are referred for specialist care if indicated and prioritizing specialist-PCP communication may be an important component of pediatric asthma care that was not addressed in the early years of Massachusetts Medicaid ACOs.

Structural contributors to fragmentation in the U.S. health care system may make it difficult for health care providers to meet caregivers' and children's needs for asthma care. Value-based care models theoretically improve care coordination and management, including expanded capacity to identify and address HRSN as part of care management. Some prior studies have shown improvement in pediatric asthma outcomes, such as reducing ED visits and hospitalization rates, associated with ACO implementation, but these effects were generally seen in pediatric-only ACOs or ACOs that had programs specifically targeting pediatric asthma quality of care (7, 39). Prior studies conducted by our team suggest that there were limited reductions in disparities between Medicaid-insured and privately insured children in asthma quality measures, ED visits, or hospitalizations in the three years following Massachusetts Medicaid ACO implementation (21). These results are consistent with the current study's findings that caregivers did not perceive changes in asthma care following ACO implementation. Possible explanations for the lack of measured or perceived effect of the Massachusetts Medicaid ACOs on pediatric asthma care and outcomes include a lag between implementation and when effects are noted, as has been shown in other ACO studies (40) and the potential for a greater focus on adult populations with multiple chronic diseases where there is more opportunity to improve quality and reduce costs, particularly in the early years of implementation (41). Understanding how pediatric asthma care quality, outcomes, and disparities evolve over time as the new Medicaid ACOs mature may be warranted, as well as exploration of other health care reform that may be needed for ACOs to meet their full potential for addressing structural barriers to optimal asthma care.

This study should be considered in the context of its strengths and limitations. Interviews were conducted in 2023, several years after the ACOs were first implemented, which may have made it difficult for caregivers to recall care prior to ACO implementation or their child may not have received care for asthma prior to ACO implementation. It is also possible that caregivers might feel that care has improved but not be aware of processes that may have changed because of ACO implementation. The ACOs might have also achieved a level of maturity and stability where change is less apparent. However, major changes in large complex healthcare systems generally take years to fully implement and the COVID-19 pandemic's shocks to the healthcare system likely delayed the maturation process. Participants were purposively sampled from ACOs with varying organizational characteristics, aiming to include a range of experiences and perspectives in the study and are not intended to be fully representative of the entire Medicaid-insured pediatric population. Although interviews were conducted until data saturation was achieved, inclusion of participants from additional ACOs in Massachusetts or other practices within the target ACOs, might identify additional themes. Parents' perceptions may differ from other stakeholders' perspectives and experiences; exploring PCPs', pulmonary and allergy specialists', and insurers' perspectives could provide a more holistic view of Medicaid ACOs' effects on pediatric asthma care.

In conclusion, this study of caregivers of children with asthma whose PCP's practice was part of a Massachusetts Medicaid ACO suggests that caregivers may have continued to face challenges in asthma care access, quality, and equity following Medicaid ACO implementation. Medicaid ACOs in Massachusetts entered new contracts in 2023 that differ from the 2018–2022 contracts in ways that may differentially affect pediatric asthma care. Examples include introduction of universal sub-capitation reimbursements at the practice level and greater emphasis on pediatric care and behavioral health care and may warrant further study of the ACOs' effects on pediatric asthma care and outcomes (42). This study's results suggest that there may be interventions Medicaid ACOs could consider at health care system, community, practice, and individual levels to improve pediatric asthma care quality and outcomes. Integrating primary and specialty care within primary care practices, much like the behavioral health integration model; systematizing asthma education that meets caregivers' and children's needs, improving primary care continuity if indicated, increasing advocacy for healthy indoor and outdoor environments, and assuring systematic responses to positive HRSN screens with resources may help to further address the needs identified. Although many of the recommendations participants made, and those developed inductively in data analysis, resonate with prior research (43–47), the persistence of these issues and the potential for the care transformation brought about by value-based Medicaid ACOs is a contribution to our understanding of potential pathways to optimize delivery of high quality equitable care for the large number of Medicaid-insured children with poorly controlled asthma.

## Data Availability

Data requests will be considered if application and proposed use are within the scope of the informed consent and we can be sure to maintain confidentiality consistent with our IRB approved protocol. Requests to access the datasets should be directed to Sarah at Goff sgoff@umass.edu.
