# Supplementary material for: Parent-identified opportunities for improving asthma care for children insured by Medicaid following implementation of statewide Medicaid Accountable Care Organizations in Massachusetts
Source: Front Allergy. 2025 Nov 24;6:1695447. doi: 10.3389/falgy.2025.1695447 (PMC12682864; doi:10.3389/falgy.2025.1695447)
Supplement: Supplementary file 1 [file Table1.docx]

**Table 1: Participant and Practice Characteristics**

| **Participants (n=26)** | **n (%)** |
| --- | --- |
| **Caregiver Age**  < 30  30 - 39  40- 49  ≥ 50 | 3 (11.5)  14 (53.8)  4 (15.4)  5 (19.2) |
| **Caregiver Gender**  Male/Other  Female | 1 (3.8)  25 (96.2) |
| **Caregiver Race/Ethnicity ***  White  Black  Latino/Hispanic  Other (multi-racial) | 9 (34.6)  7 (26.9)  10 (38.5)  1 (3.8) |
| **Age of Child**  <5  5-9  10-14  15-17 | 3 (11.5)  12 (46.2)  8 (30.8)  3 (11.5) |
| **Regions Represented (# of practices)**  Western (10)  Central (1)  NorthEast (1)  SouthEast (3) | 15 (57.7)  1 (3.8)  3 (11.5  7 (26.9) |
| **Number of practices per ACO**  A (7)  B (4)  C (4) | 15 (57.7)  6 (23.1)  5 (19.2) |
| ***** Not mutually exclusive categories |  |
