# Supplementary material for: Parent-identified opportunities for improving asthma care for children insured by Medicaid following implementation of statewide Medicaid Accountable Care Organizations in Massachusetts
Source: Front Allergy. 2025 Nov 24;6:1695447. doi: 10.3389/falgy.2025.1695447 (PMC12682864; doi:10.3389/falgy.2025.1695447)
Supplement: Supplementary file 2 [file Table2.pdf]

Table 2: Supplemental Major Themes, Subthemes and Illustrative Quotes

Theme	Asthma Disparities Framework	Quotes
**1) Perceived lack of changes in asthma care related to Medicaid ACO implementation**	Health Policies-Payment Models	*I knew that [name] practice was in an ACO just based off of like the information that I received from MassHealth, but I didn't really understand like what that means or why that's important (03-05; Private Practice; Southeast; Black; not Hispanic or Latino)*
**2) Insurance coverage influence on asthma care**	Health Policies-Insurance Status	*MassHealth['s] unwillingness to pay for things that your kid really needs [makes asthma care more challenging]. (03-06; Private Practice; Western; White; not Hispanic or Latino)*
**3) Perceptions of primary care asthma management**		
Communication	Clinician Factors-Patient-Provider Communication	*So they explain [the asthma action plan] very well to me. They even write it down for me and they call me after the fact and then they even check up and then they make sure that I'm following the instructions that were given to me. So they do that very well also. (01-03; Private Practice; Western; White; not Hispanic or Latino)*
Workforce diversity	Health Care Operations-Workforce Diversity	*…both of my son's doctors, and this is no offense to them, but they're both like, I'm assuming here, but upper class White women, and I'm a Black woman, I'm a single mom. It's like what would they be able to tell me that would help in those [HRSN] situations? Not to say that they can't empathize, but I would need more than just empathy… What real help is there? (03-05; Private Practice; Southeast; Black; not Hispanic or Latino)*

<u>Continuity of care</u><br><br><br><br><br><br><br><br><br><u>Perceptions of PCP support in managing asthma</u>	Processes of Care-Quality of Care<br><br><br><br><br><br><br><br><br>Health Care Operations-Evidence Based Practice	*Before connecting with the lung specialist, I really didn't feel like I had anyone to go to unless I was making an [urgent] appointment [with the PCP...] But it also never was the same provider [at PCP's office]. I could see one nurse practitioner one time and then a different one the next time. So never really the same doctor that was listening to my daughter's lungs. Whereas now that we just have the asthma specialist, we just go to her for any of those respiratory concerns. (03-12; Private Practice; Southeast; White; not Hispanic or Latino)*<br><br>*I feel like… [pediatrician] helped me a lot [with asthma care]. Because she also takes care of my other child too as well. So, yeah, she helped me a lot. (3-15; Private Practice; Western; Black; not Hispanic or Latino)*<br><br>*[Asthma specialist referral is] something that has not been brought up. Like, even when I started seeing [medical practice], they never brought this up. So it's like I didn't know, I thought I'd just see his doctor about it and that's it. And if it's an emergency go to the ER, that's the only thing that I'm aware of. (01-05; CHC owned by a hospital; Western; Race Not Reported; Hispanic or Latino)*<br><br>*I'd like them to have referred him for better care ... something to improve my son's asthma... I did ask [for a referral], and they said they would see how he did. If he wasn't better in a few weeks, that I should take him to ER and then they would see. It was always like that. (04-02, CHC; Western; race not reported; Hispanic or Latino)*
**4) Perceptions of specialist care for asthma**<br><br><u>Satisfaction with care and care coordination</u>	Clinician Factors-Patient-Provider Communication	*[The communication] is vastly different [between the specialist and PCP], like I know that [the specialist] has called me before and then after phone call, she'd send me like a typed out message just recapping everything we discussed, because I have like the baby and then the other two. So she's like, all right, I'm going to end this. So I'm going to send you a message just so*

		you can read it and have it all in front of you. (03-12; Private Practice; Southeast; White; not Hispanic or Latino)
Access	Process of Care –Access to Treatment	*That's the one downfall about specialists is that their schedules are usually so booked up… even when it came down to booking [child's] follow-up appointment for December when we initially tried to book it, they're like, oh, pulmonologist is booked out to June of next year. And I said, 'Well, that doesn't work for her care so we have to figure something out.' So, I actually had to reach out to her pulmonologist and she was the one that [said], 'Hey, I'm scheduling, it looks like I actually have a free slot here. I need her to be seen on this date.' (03-14; Private Practice; Southeast; Black; not Hispanic or Latino)*
**5) Influence of health-related social needs on pediatric asthma care and outcomes**	Individual/Community - Social/ Environmental	*The only thing that makes it difficult to manage… my kids health care is it's either I lose those days paid or my kids health gets worse… I used to work three jobs a year and a half ago. [...] I just couldn't do it anymore with her being sick… So I just quit my job. I quit all three jobs and I found a job like closer up the street from my house. So it took a lot like this financial toll... (03-07; Private Practice; Southeast; Black; not Hispanic or Latino)*<br><br>*I would say the bulk of the appointments have more so been focused [on] physical symptoms and how he's doing overall. But I've never been asked [about HRSN]… Not to say that it can't be brought up in the doctor's office, but I also feel like what can they actually do or what would they tell me? (03-05; Private Practice; Southeast; Black; not Hispanic or Latino)*<br><br>*It's basically just me [taking care of child with asthma]. We don't have any family around here and doctors they're not as personable as they used to be. (03-06; Private Practice; Western; White; not Hispanic or Latino)*<br><br>*I don't know if environmental racism is a term, but that's a big issue. I felt like my landlord was kind of taking advantage of the situation and felt like I couldn't do anything about it… I feel like landlords… don't actually want to do anything, keep their own rules. Like you have a policy about not smoking*

		but you're not enforcing it. (03-05; Private Practice; Southeast; Black; not Hispanic or Latino)
**6) Suggestions for improving pediatric asthma care**		*[Healthcare team] staying in contact [would improve asthma care]… because asthma is I feel like a lifelong thing… if you have diabetes, you go on a regular basis, and I don't think that that happens [with asthma], and I think it should. (03-06; Private Practice; Western; White; not Hispanic or Latino)*<br><br>*… besides having asthma specialists [in PCP office]… maybe the doctors [PCPs] can get a little bit of training… in that area [then]…, if the parents doesn't want to see an asthma specialist it might be helpful that they would have maybe a little bit of background… like a quick little course on what an asthma specialist would do. (03-02; Private Practice; Northeast; Race Not Reported; Hispanic or Latino)*<br><br>*[responding to a question about community health workers]… I never heard of [a community health worker... [I'm] actually shocked to know [they exist] because I never knew that [was a possibility]... I'd love for somebody to come in here and be like, this is what [to do] -- if they see something that I didn't know about." (03-10; Private Practice; Western; White; not Hispanic or Latino)*
		*Study ID#; practice type; geographic region; race of participant; ethnicity of participant
